# The role of isothiocyanate-rich plants and supplements in neuropsychiatric disorders: a review and update

**DOI:** 10.3389/fnut.2024.1448130

**Published:** 2024-09-30

**Authors:** Monica Ramakrishnan, Jed W. Fahey, Andrew W. Zimmerman, Xinyi Zhou, Anita A. Panjwani

**Affiliations:** ^1^Department of Nutrition Science, College of Health and Human Sciences, Purdue University, West Lafayette, IN, United States; ^2^Department of Medicine, Johns Hopkins School of Medicine, Baltimore, MD, United States; ^3^Department of Pharmacology and Molecular Sciences, Johns Hopkins School of Medicine, Baltimore, MD, United States; ^4^Department of Psychiatry, Johns Hopkins School of Medicine, Baltimore, MD, United States; ^5^Institute of Medicine, University of Maine, Orono, ME, United States; ^6^Department of Pediatrics, UMass Chan Medical School, Worcester, MA, United States; ^7^Center on Aging and the Life Course, Purdue University, West Lafayette, IN, United States

**Keywords:** sulforaphane, glucosinolate, cruciferous, neurodevelopmental, neurodegenerative, neuroinflammation

## Abstract

Neuroinflammation in response to environmental stressors is an important common pathway in a number of neurological and psychiatric disorders. Responses to immune-mediated stress can lead to epigenetic changes and the development of neuropsychiatric disorders. Isothiocyanates (ITC) have shown promise in combating oxidative stress and inflammation in the nervous system as well as organ systems. While sulforaphane from broccoli is the most widely studied ITC for biomedical applications, ITC and their precursor glucosinolates are found in many species of cruciferous and other vegetables including moringa. In this review, we examine both clinical and pre-clinical studies of ITC on the amelioration of neuropsychiatric disorders (neurodevelopmental, neurodegenerative, and other) from 2018 to the present, including documentation of protocols for several ongoing clinical studies. During this time, there have been 16 clinical studies (9 randomized controlled trials), most of which reported on the effect of sulforaphane on autism spectrum disorder and schizophrenia. We also review over 80 preclinical studies examining ITC treatment of brain-related dysfunctions and disorders. The evidence to date reveals ITC have great potential for treating these conditions with minimal toxicity. The authors call for well-designed clinical trials to further the translation of these potent phytochemicals into therapeutic practice.

## 1 Introduction

**Figure 1:**
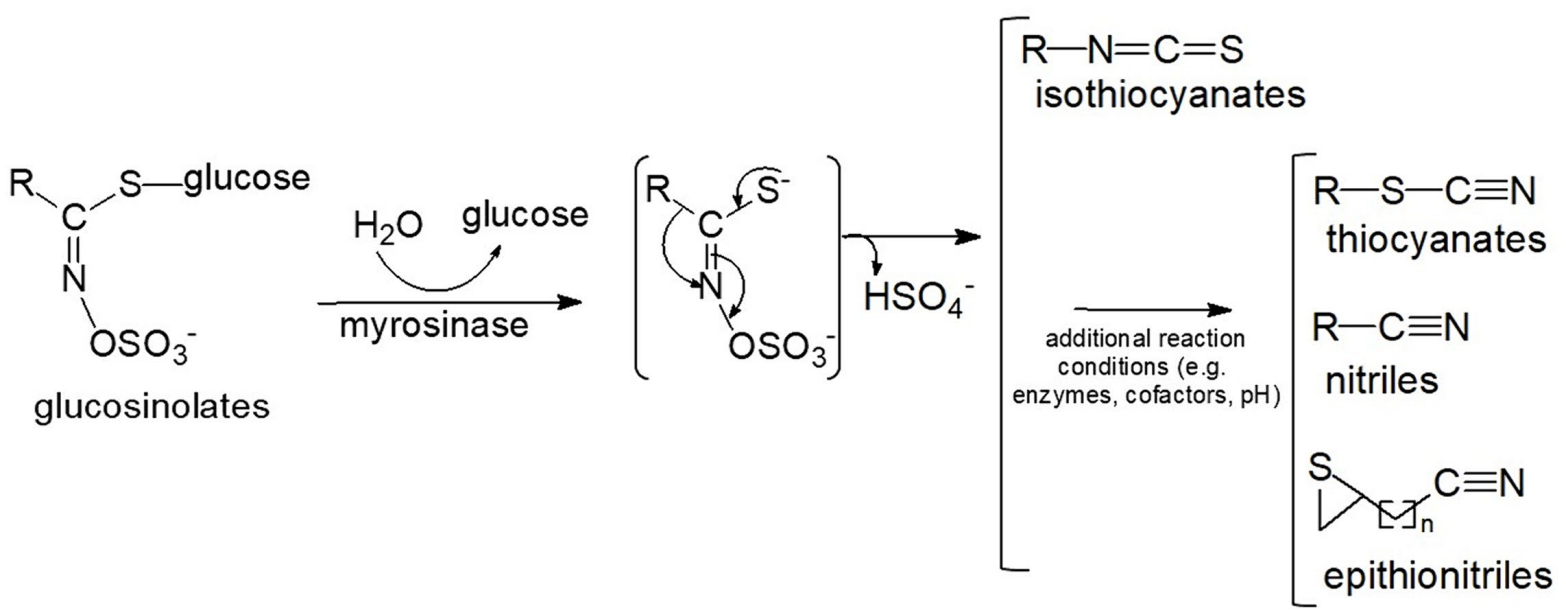
Conversion of glucosinolates to isothiocyanates and other reaction products. The myrosinase reaction produces an unstable intermediate which then non-enzymatically rearranges to isothiocyanates, or, in the presence of additional enzymes, cofactors and favorable conditions (e.g., low pH) can form a variety of alternative end-products including thiocyanates, nitriles, and epithionitriles (8).

Isothiocyanates (ITC) found in cruciferous and related vegetables (e.g., broccoli and cabbage), exhibit diverse properties such as antibacterial, antifungal, antioxidant, and cytoprotective effects. They are produced as a result of enzymatic activity of myrosinase on glucosinolates (ITC precursors) present in plant cell vacuoles (Figure 1). Myrosinase is compartmentalized in cells of the same plant tissues and released upon cell lysis (e.g., chewing or wounding) (1). Additionally, microbiota in the gastrointestinal tracts of animals also contribute to this conversion. However, intraindividual differences in microbial conversion vary greatly, resulting in highly variable bioavailability (2). The formation of isothiocyanates from glucosinolates is also influenced by several other factors (3), including pH value, ferrous ions, and specifier proteins such as epithiospecifier protein (ESP), nitrile-specifier protein (NSP), and thiocyanate-forming protein (TFP). ESP, NFP and TFP can lead to the production of epithionitriles or nitriles upon the hydrolysis of glucosinolates in plant tissues (4). Additionally, glucosinolates undergo enzymatic conversion to amines (3, 5). The incomplete or variable conversion of glucosinolates to isothiocyanates may account for differing effects observed in various studies. Additional factors include genetic variability in the glutathione S-transferase M1 gene that could affect the metabolism of SF (6).

Sulforaphane (SF), derived from its glucosinolate precursor, glucoraphanin, is produced in greatest relative abundance in the cruciferous species *Brassica oleracea* var. *italica* (broccoli) as well as in a rangeland weed (*Cardaria draba*), some red cabbage (*B. oleracea var. capitata*) and red kale (*B. oleracea var. capitata*) sprouts. Sulforaphane stands out as a highly studied and promising therapeutic agent currently undergoing preclinical and clinical evaluation of a multitude of diseases and disorders (7). Apart from cruciferous vegetables, which are most familiar to human consumers, 15 other plant families produce over 120 different glucosinolates (8–10). A noteworthy example includes the tropical tree vegetable, *Moringa oleifera*, which is gaining wide recognition for its exceptional nutrient profile along with its cytoprotective properties; these properties are attributable in part to its primary ITC, moringin, derived from the glucosinolate glucomoringin (11). While there are documented toxic effects associated with the overconsumption of cruciferous and related vegetables, such as cabbages rich in indoles and thiocyanates and the presence of plant-derived toxins, they have been safely consumed by many people across the globe as part of a well-balanced diet (12).

Chronic oxidative stress, along with neuroinflammation, plays a central role in the pathogenesis of numerous neuropsychiatric disorders. For instance, in several clinical and preclinical studies, SF counteracts several molecular abnormalities associated with autism spectrum disorder (ASD) that address reduced antioxidant capacity, enhanced oxidative stress, deficient glutathione synthesis, mitochondrial dysfunction, increased lipid peroxidation, and neuroinflammation (2). SF’s ability to readily cross the blood–brain barrier likely enables it to exert its clinical neurological effects (1).

A key mechanism of SF involves the activation of the nuclear transcription factor Nrf2 (nuclear factor erythroid 2–related factor 2), which orchestrates a suite of cytoprotective responses. SF binds with Keap1 (Kelch-like ECH-associated protein 1), causing its dissociation from Nrf2. Nrf2 is then free to translocate into the nucleus and induce the transcription of phase 2 detoxification enzyme genes (2). SF also induces anti-inflammatory, heat shock-response (HSR)-inducing, and histone deacetylase (HDAC) inhibiting responses, contributing to its multifaceted cytoprotective effects (2, 13).

Evidence for the effects of ITC from plants other than broccoli, such as *Moringa oleifera* and *Raphanus sativus* (radish), on chronic diseases including cancer, neurologic disorders, and cardiovascular diseases has been reviewed (14–25). We seek herein to review the effects of ITC and ITC-rich plants on brain-related disorders. We include reports on both clinical and pre-clinical studies demonstrating the potential of ITC to ameliorate these disorders. Our focus is on studies published from 2018 to 2023, and serves as an update to our previous review conducted in 2018, though we do reference some studies prior to this period to provide context (26).

## 2 Neurodevelopmental disorders

Recent clinical and preclinical studies have explored interventions in neurodevelopmental conditions using ITC. These studies mark the initial attempts to address disorders with disparate causes and pathophysiology through targeting potentially common pathways with these phytochemicals.

### 2.1 Autism spectrum disorder

Autism spectrum disorder (ASD) is a complex and heterogeneous neurodevelopmental disorder characterized by deficits in social communication and restricted, repetitive behaviors (27). The current prevalence of ASD among 8-year-old children in the US is 1 in 36 (28), with over 600,000 new cases diagnosed annually worldwide (29). Currently, there are no FDA-approved pharmacological treatments for the core features of ASD. In this review, we report 9 clinical studies from 2014 to 2023, including 6 randomized controlled trials (RCTs), 1 non-randomized single arm clinical trial, and 1 case study related to the benefits of ITC on ASD (Table 1). We also report findings of ITC treatment in five preclinical studies, including 4 *in vivo* and 1 *in vitro* models of ASD.

**Table 1:** Clinical studies reported the effects of sulforaphane or glucoraphanin treatments on behavioral and health outcomes in individuals with ASD.

Author, Year (Study type)	Study population	Compound(s) delivered	Treatment and dose	Duration of intervention	N	Results
Singh et al., 2014 (30) (RCT)	Males (13–27 years old) with moderate to severe ASD	SF	50–150 μmol SF/day dosed by body wt.	18 weeks	44	Significant improvement of behavior scores [*p* < 0.001 (ABC) and *p* = 0.017 (SRS)].
Evans & Fuller, 2016 (37) (Case study)	Males (3–33 years old) enrolled from a public ASD forum	GR + myrosinase	Myrosinase Activated BroccoMax® containing a standardized GR content (the manufacturer claimed an *in vitro* yield of *ca.* 8 mg of SF, but SF delivery was not validated by the study authors)	≤28 weeks	6	Among children: Improvements in 69% of behavioral attributes, with substantial improvement in 29% (ABC and other unknown measures). Among adults: Improvement of 100% of behavioral attributes in the SRS, with substantial improvement in 59% (ABC and other unknown measures).
Bent et al., 2018 (33) (RCT)	Children and young adults (5–22 years old) with ASD and related neurodevelopmental disorders	GR + myrosinase	6–15 Avmacol tablets dosed at 222–555 μmol GR daily depending on body wt.	12 weeks	15	Improvement in mean behavior scores [ABC (ns) and SRS (*p* = 0.03)]. Urinary metabolites correlated with symptom amelioration.
Momtazmanesh et al., 2020 (35) (RCT)	Children (4–12 years old) with ASD	SF	50 μmol of SF/day for body weight lesser than 45 kg and 100 μmol of SF/day for 45–90 kg body wt.	10 weeks	60	Significant improvements in irritability (*p* = 0.001) and hyperactivity (*p* = 0.015), but no improvement in lethargy/social interaction, stereotypy, inappropriate speech (ABC) and frequent adverse events.
Zimmerman et al., 2021 (32) (RCT)	Children (3–12 years old) with ASD	GR + myrosinase	Avmacol equivalent to 2.2 μmol/kg/day SF	36 weeks	60	Significant improvement in scores with a non-randomized analysis of SF exposure duration (ABC *p* < 0.001), but no effects on SRS or OACIS. Significantly lower oxidative stress at 15 weeks (*p* < 0.05 for glutathione redox states). Significantly lower expression of IL-6 (*p* = 0.006) and TNF-α (*p* = 0.01) at 15 weeks but higher IL-1β expression at 7 weeks (*p* = 0.03) and 30 weeks (*p* = 0.03, 0.04). Lower heat shock protein (HSP70) expression correlated with symptom amelioration (ABC; *p* < 0.05) at 15 weeks. Improved mitochondrial function (*p* < 0.05 for increased ATP-Linked Respiration) and improvement correlated with symptom amelioration (ABC).
Ou et al., 2022 (34) (RCT)	Children (3–15 years old) with ASD	GR + myrosinase	Avmacol (2–8 tablets) daily depending on body wt. (4.5–60 kg)	12 weeks	110	Significant improvements of autism features [*p* < 0.001 (CGI-I) and *p* < 0.002 (OARS-4)], but no significant effects on behavior [SRS (*p* = 0.885), ABC (*p* = 0.706) and RBS-R (*p* = 0.171)]. Improvements higher in participants older than 10 years of age and effects spanned range of intelligence.
Magner et al., 2023 (38) (RCT)	Children (3–7 years old) with ASD	GR + myrosinase	Laboratory preparation of broccoli and red radish sprout powder mix containing 50 μmol of SF per day	36 weeks	28	No significant improvement in behavior and general level of ASD (ABC, SRS-2 and ADOS-2).
Yang et al., 2023 (36) (Single arm clinical trial)	Boys (4–7 years old) with ASD (n = 6) or healthy controls (n = 11)	GR + myrosinase	Avmacol (2–8 tablets) daily depending on body wt. (4.54–58.97 kg)	12 weeks	17	Significant improvement in verbal or non-verbal communication [*p* < 0.05 (OARS-4)], but not in social interaction and repetitive/ritualistic behaviors. No gut microbial diversity associated with treatment. Correlation of 35 gut microbiome abundance alterations with symptoms of ASD.
Buyske, 2024 (RCT)	Young adults (13–30 years old) with ASD	GR + myrosinase	Avmacol daily; 1.5 μmol GR/kg body wt.	18 weeks	45	None reported yet; NCT02677051
Politte, 2024 (RCT)	Young men (13–30 years old) with ASD	GR + myrosinase	Tablets containing 125 mg broccoli seed extract and 50 mg broccoli sprout extract, equating to *ca.* 15 μmol SF per tablet; dose range: 3–8 tabs/d. based on the participant’s wt.	12 weeks	48	None reported yet; NCT02909959

N, Number of participants; RCT, Randomized Clinical Trial; ASD, Autism Spectrum Disorder; SF, Sulforaphane; Wt., Weight; ABC, Aberrant Behavior Checklist; SRS, Social Responsiveness Scale; GR, Glucoraphanin; μmol, Micromole; mg, Milligram; kg, Kilogram; OACIS, Ohio Autism Clinical Impressions Scale; IL-6, Interleukin-6; TNF-α, Tumor Necrosis Factor-alpha; IL-1β, Interleukin-1 beta; ATP, Adenosine Triphosphate; CGI-I, Clinical Global Impression Scale for Improvement; OARS-4, Ohio State University (OSU) Autism Rating Scale-Diagnostic and Statistical Manual of Mental Disorders (DSM)- Fourth edition (IV); RBS-R, Repetitive Behavior Scale-Revised; ADOS-2, Autism Diagnostic Observation Schedule, Second Edition.

#### 2.1.1 Clinical studies

The first randomized controlled trial conducted in 2014 provided compelling evidence of the therapeutic potential of SF in the management of core features of ASD (NCT01474993) (30). Over an 18-week period, SF treatment (2.2 μmol/kg/day SF) significantly improved social interaction, reduced aberrant behavior, and enhanced verbal communication in autistic males aged 13–27 years. These effects returned to near baseline after a 4-week washout period. Further, responders who continued to consume dietary supplements containing glucoraphanin (GR), the glucosinolate precursor of SF reported sustained benefits for 3 years (31).

Since this first RCT, several clinical studies have attempted to replicate these findings. While the methodology has varied, most studies reported positive results though improvements were not as profound as in the initial study (32–37). The same group conducted another RCT in children aged 3–13 years, using GR plus myrosinase (2.2 μmol/kg/day SF), which showed mixed results (32). While clinical ratings did not significantly differ between the intervention and placebo groups using the clinician-rated Ohio Autism Clinical Impressions Scale (OACIS), there were significant improvements in caregiver-reported measures of aberrant behaviors (ABC-2). In addition, reductions in inflammatory biomarkers (TNF-*α*, IL-6, IL-1β) and improvements in glutathione redox status and mitochondrial function were apparent in the intervention group compared to the placebo group. Another study tested an adjuvant treatment of SF (50 or 100 μmol of SF/day depending on body weight) with risperidone and found children in the treatment group exhibited greater improvements in irritability and hyperactivity compared to the placebo group (35). A single-arm clinical trial found minimal amelioration in both ASD-related behaviors and fecal microbiome diversity with 2–8 GR plus myrosinase tablets/day depending on body weight (36), and one study found no difference in behavioral measures between treatment and placebo groups with 50 μmol/day (38).

In evaluating the mixed findings, we observed that the two studies that showed greatest improvements delivered SF itself (the plant-derived ITC, not a synthetic version, in a supplement matrix that represented some subset of the original plant extract with its attendant phytochemical diversity) while all other studies relied on the combination of a plant extract rich in GR and co-delivering active plant-derived myrosinase (30, 35). While one of these studies used SF as an adjunctive therapy, we posit that the method of delivery, SF, may be the key to minimizing differences in bioavailability and, consequently, achieving significant behavioral improvements. In the study that did not show any difference in behavior (30), SF (45.1 μmol) may have been inactivated due to high temperatures involved in its preparation. The pharmacokinetics of ITC production from glucosinolates in the gastrointestinal system, vs. delivery of pre-formed SF, are not fully understood, but what is currently known gives credence to this conjecture. The metabolic conversion of GR to SF may vary both developmentally and between individuals. At this writing (spring 2024) there are two ongoing studies using GR + myrosinase that are yet to report their findings on treatment in individuals with ASD (NCT02677051, NCT02909959; Table 1).

#### 2.1.2 Pre-clinical studies

Interventions including oral SF-rich and glucoraphanin-rich preparations, as well as those of the plant maca (*Lepidium meyenii*), have shown promising outcomes in improving autism-associated outcomes. For example, SF treatment (282 μmol/kg/day for 7 days) reduced self-grooming/marble-burying behavior, improved social interaction, decreased T helper 17 (Th17) immune responses and oxidative stress, and increased enzymatic antioxidant defenses in an autism mouse model (39). In another study of a maternal immune activation (MIA) mouse model, GR intake (0.1% GR food pellets) of dams during pregnancy and lactation prevented cognitive and social interaction deficits in juvenile offspring and cognitive deficits in adult offspring (40). Moreover, oral administration of supplements containing GR and active myrosinase (2.2 μmol SF/kg/day for 14 days) increased mRNA levels of cytoprotective enzymes and heat shock proteins while decreasing mRNA levels of pro-inflammatory markers in peripheral blood mononuclear cells (PBMC) of patients with ASD (41). A study focusing on Nrf2 activation demonstrated SF (5 μmol incubated overnight) ameliorated inflammation and nitrative stress in ASD monocytes induced by LPS (lipopolysaccharide) (42). Finally, supplementation with maca (5 g/kg/day for 14 days)—an uncommon vegetable in the US, but one with high levels of glucosinolates—improved social deficits by upregulating oxytocinergic pathways in a valproic acid mouse model of ASD (43).

### 2.2 Schizophrenia

Schizophrenia is a progressive neurodevelopmental disorder with disruptions in cognitive functions, perceptual understanding, and executive function. Antipsychotic drugs are commonly used in the management of schizophrenia and can have long-term side effects. There have been four clinical studies—1 of which was an RCT—and 2 *in vivo* preclinical studies on schizophrenia and ITC treatment. Early intervention efforts for prodromal symptoms are being explored in patients with schizophrenia, including the incorporation of ITC like SF in the diet (44).

#### 2.2.1 Clinical studies

Several clinical studies have reported on the effects of SF treatment in schizophrenia (Table 2). In an open-phase trial, participants with schizophrenia (*N* = 7) displayed significantly enhanced accuracy in the One Card Learning Task after 8 weeks of GR (68.5 μmol/day) administration (45). Similarly, another open-label study in this patient population (*N* = 45) demonstrated that a higher dose of GR + myrosinase (507.4 μmol SF/day) for 24 weeks resulted in significantly increased superoxide dismutase activity and HsCRP levels and a significant decrease in Positive and Negative Syndrome Scale (PANSS) negative subscale scores (46). Investigators aiming to study schizophrenia first conducted a study in healthy volunteers (*N* = 9) and demonstrated elevated blood glutathione levels after a week of daily SF (100 μmol/day for 7 days) consumption (47). The investigators also measured brain metabolites through magnetic resonance spectroscopy (MRS) scanning and reported a significant increase in glutathione concentration in the hippocampus. However, a randomized controlled trial (*N* = 58) found no differences between GR + myrosinase (100 μmol SF per day for 16 weeks) treatment and placebo groups in PANSS scores and cognitive functioning among individuals with schizophrenia (48). An ongoing RCT (*N* = 480) in China is exploring add-on treatments of minocycline (200 mg) and GR (68.5 μmol SF/day for 8 weeks) with antipsychotics to patients with schizophrenia who were non-responsive to the antipsychotic drugs alone (NCT03451734) (49).

**Table 2:** Clinical studies reported the effects of sulforaphane or glucoraphanin treatments on behavioral and health outcomes in individuals with schizophrenia.

Author, Year (Study type)	Study population	Compound(s) delivered	Treatment and dose	Duration of intervention	N	Results
Shiina et al., 2015 (38) (Single arm clinical trial)	Outpatients (20–65 years old) with schizophrenia	GR	3 tablets each day equivalent to 68.5 μmol of GR/day	8 weeks	10	Significant improvement in the accuracy component of the OCLT (*p* = 0.043) post-SF treatment. CogState battery scores, PANSS total scores and serum BDNF levels were not different pre-and post-SF treatment
Zeng et al., 2024 (39) (Single arm clinical trial)	Patients (18–50 years old) with schizophrenia	SF	3 Nutramax tablets each day equivalent to 507.4 μmol of SF/day	24 weeks	66	Improvement in PANSS negative subscale (*p* < 0.001) and total scores (*p* < 0.001). No difference in PANSS positive subscale (*p* > 0.05). Increased SOD activity (*p* < 0.05) and HsCRP levels and (*p* < 0.05).
Sedlak et al., 2018 (40) (Single arm clinical trial)	Healthy volunteers (eight of them aged 21–26 years old and 1 participant aged 56 years)	SF	2 gel capsules of standardized broccoli sprout extract containing 100 μmol SF/day	7 days	9	Increased GSH in non-monocytes (*p* = 0.02) and left hippocampus (*p* = 0.041). Blood GSH levels positively correlated GSH in bilateral thalamus brain region (*p* = 0.017). Positive correlations between blood GSH and gamma-aminobutyric acid, glutamine, glutamate, and GSH in the bilateral thalamus were not significant.
Dickerson et al., 2021 (41) (RCT)	Individuals (18–65 years old) diagnosed with scizophrenia	GR + myrosinase	6 Avmacol tablets dosed at 222 μmol GR (equivalent to 100 μmol SF) per day	2 weeks of placebo and 16 weeks of SF	58	No improvement in PANSS scores and cognitive functioning (as measured by MCCB and domain cognitive scores).
Xiao et al., 2021 (42) (RCT)	First-episode schizophrenia patients with less than 25% reduction rate of PANSS score with antipsychotics treatment	GR	3 tablets of GR each day equivalent to 68.5 μmol of GR/day	8 weeks	240	None reported yet; NCT03451734

SF, sulforaphane; mg, milligrams; N, number of participants; OCLT, One Card Learning Task; PANSS, Positive and Negative Syndrome Scale; BDNF, brain-derived neurotrophic factor; SOD, superoxide dismutase; HsCRP, high-sensitivity c-reactive protein; GSH, glutathione; RCT, randomized controlled trial; MCCB, MATRICS Consensus Cognitive Battery.

#### 2.2.2 Pre-clinical studies

Preclinical studies have provided further insights into the potential benefits of SF in schizophrenia. For example, offspring from pregnant mouse dams subjected to maternal immune activation, fed with a GR-rich diet (~2.3 μmol/kg), showed protection from cognitive defects (50). Similarly, mice repeatedly exposed to phencyclidine demonstrated attenuated cognitive deficits when pretreated with a GR-containing diet (~2.3 μmol/kg) (51).

### 2.3 Cerebral palsy

Cerebral palsy is a neurological disorder characterized by difficulties in movement, balance, and posture due to weakness or impairment in muscle control and is the most common motor disability in childhood. It is usually associated with pre-or perinatal brain injury or maldevelopment. One *in vivo* study of cerebral palsy with SF treatment has been published. In this preclinical study, rat offspring were exposed to prolonged intrauterine ischemia *in utero*. Offspring born to dams given a diet supplemented with broccoli sprouts (200 mg/day from the beginning of the third trimester to postnatal day 14) had a reduced likelihood of neurocognitive impairment compared to those born to dams that did not receive the treatment (52).

### 2.4 Fetal alcohol syndrome disorders

Fetal alcohol syndrome disorder (FASD) encompasses a spectrum of conditions resulting from prenatal alcohol exposure, often presenting as a combination of physical, behavioral, and learning difficulties. We have identified two *in vivo* models and one *in vitro* experiment examining the effects of SF on mechanisms involved in FASD, including cell death and oxidative stress. Mouse embryos exposed to ethanol *in vivo* and treated with SF (1 μM for 24 h) displayed reduced apoptosis in neural crest cells compared to untreated exposed mice (53). The reported preventive mechanism of SF against ethanol-induced apoptosis was inhibition of HDAC and the subsequent elevation of histone acetylation in the cells (53). Inhibition of HDAC causes histone hyperacetylation, resulting in neuroprotective effects (54). Furthermore, pharmacological blockade of catalase or Nrf2 activity intensified ethanol intoxication in mice, and 28.2 μM /kg/day SF (a potent Nrf2 inducer) treatment for 5 days, attenuated these effects (55), leading the study’s authors to suggest that Nrf2 activation may serve as a potential therapeutic strategy for preventing acute alcoholism by regulating catalase-mediated ethanol oxidation.

### 2.5 Fetal hypoxic–ischemic brain injury

Fetal hypoxic–ischemic encephalopathy (HIE) due to placental insufficiency is characterized by low blood and oxygen supply to the fetus. This can result in both intrauterine growth restriction and brain injury. Despite the severity of this condition, research on potential interventions remains limited. One *in vitro* study highlighted the potential efficacy of SF in mitigating the effects of placental insufficiency on neuronal cells (56). In this study, the dose response of SF (0–200 μM) within different brain cell types was examined. A significant protective effect of SF was documented at a concentration of 2.5 μM in astrocytes and co-cultures of multiple cell types but not in neurons alone. Toxicity was not observed until concentrations reached ≥100 μM in astrocytes and ≥ 50 μM in co-cultures under oxygen/glucose deprived conditions.

## 3 Neurodegenerative disorders

Since oxidative stress and inflammation play a large role in the pathophysiology of neurodegenerative disorders, the ITC SF and moringin, among other Nrf2 inducers, have garnered attention for their potential to alleviate various symptoms. These disorders include Alzheimer’s disease, Parkinson’s disease, amyotrophic lateral sclerosis, Huntington’s disease, and multiple sclerosis, among others (57). While there are only a couple of clinical studies examining the effects of ITC in neurodegenerative disorders, several preclinical studies support the benefits of these phytochemicals (58).

### 3.1 Alzheimer’s disease

Alzheimer’s Disease (AD), the most common form of dementia, is estimated to affect 6.93 million people in the U.S. as of 2024 and is projected to reach 13.85 million by 2060 (59). ITC has shown promise in AD by preventing tau and amyloid-beta (Aβ) accumulation, two notable markers of AD. Abnormal tau builds up in memory-related brain regions, while Aβ forms plaques between neurons (60). To date, there have been 15 *in vivo*, 6 *in vitro*, and 1 *in silico* AD models studied with ITC.

SF has shown promising effects in mitigating AD pathology in several preclinical studies. Both mouse primary cortical neurons (10 or 20 μM for 3 or 6 h) and a triple-transgenic AD mouse model treated with SF (56.4 or 282 μmol/kg/day for 6 days/week for 8 weeks) exhibited enhanced brain-derived neurotropic factor (BDNF) expression, crucial for neuronal survival and synaptic plasticity, suggesting a potential epigenetic mechanism for SF’s neuroprotective effects (61). Additionally, SF administered at 28.2 μmol/kg/day for 4 months (62), 141 μmol/kg/day for 80 days (63), or 141 μmol/kg/day for 5 months (64) ameliorated spatial cognitive impairment, reduced Aβ plaques, and regulated specific HDACs, resulting in diminished plaque burden in other AD mouse models (62–64). Changes in memory consolidation and spatial learning were also induced by SF (5 μM) in adult mice (65). A mechanistic study revealed that SF (56.4 or 282 μmol/kg/day for 6 days/week for 8 weeks) cleared Aβ and tau accumulation by increasing levels of a heat shock protein (HSP70) and co-chaperone (CHIP), and mitigated memory deficits in a triple transgenic mouse model of AD (66). SF (141 or 282 μmol/kg/day for 4 months) has also been shown to protect against Aβ-induced neurotoxicity in primary mouse neurons and suppress tau protein phosphorylation in a transgenic AD mouse model (67). Moreover, the prophylactic mechanisms of SF (141 mg/kg/day for 2 weeks) have been shown to protect against LPS-induced prefrontal cortex-related recognition memory impairment in mice by improving recognition memory and reducing neuroinflammation, oxidative stress, neurodegeneration, Aβ accumulation, and microglial activation (68).

Molecular mechanisms underlying the neuroprotective effects of ITC have also been studied *in vitro*. SF (1.25 or 2.5 μM treated for 48 h) upregulated Nrf2 expression and facilitated Nrf2 nuclear translocation by reducing DNA methylation of the Nrf2 promoter in a cellular AD model (69). Furthermore, hydrogen sulfide (H_2_S)-releasing hybrids combining rivastigmine with either SF or its metabolite erucin [4-(methylthio) butyl ITC; 5 μmol treated for 24 h] exhibited protective effects against LPS-induced microglia inflammation and increased the expression of antioxidant proteins like glutathione in human neuronal (SH-SY5Y) cells (70). Examining yet another mechanism, SF (0.03 to 3 μM for 60 min) demonstrated six-fold greater potency against *β*-site amyloid precursor protein cleaving enzyme 1 (BACE1), a rate-limiting enzyme in Aβ production, as compared to resveratrol and quercetin *in silico* (71). Other preclinical investigations demonstrated positive benefits of SF on AD by attenuating Aβ oligomers-mediated reduction of phagocytic activity (5 μM for 24 h) (72), reducing streptozotocin-induced cognitive deficits (141 or 282 μmol/kg/day for 6 weeks) (73), decreasing neuroinflammation and inhibiting tau phosphorylation (1 or 2 μM for 1 h and stimulated with LPS for 23 h) (73), and reducing Aβ production (226 μmol/kg three times a week for 4 weeks) (74) in *in vivo* and *in vitro* AD models.

In addition to SF, other ITC and ITC-rich plants have also demonstrated consistent benefits in ameliorating AD-related pathology. Rats supplemented with *Moringa peregrina* (150 mg/kg/day for 2 months), rich in the ITC moringin, exhibited significant enhancement in short-term and long-term memories, along with increased BDNF, glutathione, and glutathione peroxidase (GPx), and decreased oxidized glutathione in the hippocampus (75). Leaf extracts of the widely consumed *Moringa oleifera* (400 mg/kg/day for 4 months) improved AD-related pathology, reduced Aβ burden, and enhanced synaptic plasticity in mice (76). Similar findings were observed in rats along with reduced homocysteine-induced tau hyperphosphorylation with a *Moringa oleifera* dose of 200 or 400 mg/kg/day for 14 days (77). Additionally, supplementation with *Moringa oleifera* (1, 5% or 10% in diet for 7 or 14 days) in mice mitigated scopolamine-induced spatial memory deficits, restored cholinergic transmission, and maintained neuronal integrity (78). Pretreatment with moringin conjugated with *α*-cyclodextrin (0.5 μM for 24 h) in an AD cell model reduced gene expression related to autophagy and mitophagy (79). Finally, a study of mice treated with 6-MSITC treatment [6-(methylsulfinyl) hexyl isothiocyanate; 24.3 μmol/kg/day for 10 days] from *Wasabia japonica*, or wasabi, demonstrated attenuated neuroinflammation, memory impairments, inhibited apoptosis, and oxidative stress (80).

### 3.2 Parkinson’s disease

Parkinson’s disease (PD), characterized by dopaminergic neuron loss, involves neuroinflammation, oxidative stress, mitochondrial dysfunction, and protein aggregation. One clinical study has been reported on the effects of sulforaphane on PD, while preclinical investigations examining the effects of ITCs on PD include 10 *in vivo* and three *in vitro* studies.

#### 3.2.1 Clinical studies

A self-experimented clinical study was conducted in individuals diagnosed with Parkinson’s disease using broccoli seed tea (25–45 μmol SF/day for at least 4 weeks, with weekly administration in the first week and up to twice per week thereafter) rich in SF intended to induce Nrf2 (81). Although statistical significance is uncertain as *p*-values were not provided, the findings revealed the tea reduced non-motor symptoms, including fatigue, constipation, and urinary urgency, while not affecting motor symptoms (Table 3). Currently, there is an ongoing phase 2 placebo-controlled randomized clinical trial (NCT05084365) examining the effect of SF on PD in 100 participants.

**Table 3:** A clinical study reported the effects of glucoraphanin + myrosinase compound on non-motor symptoms in individuals with PD.

Author, Year (Study type)	Study population	Compound(s) delivered	Treatment and dose	Duration of intervention	N	Results
Wright, 2024 (74) (Case study)	Individuals with Parkinson’s Disease	GR + myrosinase	30 to 40 mL of broccoli seed tea containing 25–40 μmol SF once per week during the first week with an option to increase to twice per week from the second week	4 weeks	17	Improvement in the non-motor symptoms but no change in motor symptoms

GR, glucoraphanin; SF, sulforaphane; N, number of participants; ml, milliliters; g, grams; μmol, micromoles.

#### 3.2.2 Preclinical studies

Several animal studies have also outlined the neuroprotective effects of SF in PD. One study demonstrated that SF treatment (0.05 μM for 24 h) reduced dopamine-induced cell death and restored mitochondrial membrane potential in a knockout mouse model (82). In another PD mouse model, SF (282 μmol/kg once in 2 days for 60 days) inhibited rotenone-induced deficiencies in locomotor activity and dopaminergic neuronal loss (83). Further, in MPTP (1-methyl-4-phenyl-1,2,3,6-tetrahydropyridine)-treated mice, SF treatment (56.4 μmol/kg/day for 10 days) attenuated dopaminergic neurotoxicity by activating BDNF and suppressing methyl CpG-binding protein 2 (MeCP2) (84) and activated Nrf2 (1 μM SF) (85). Sustained activation of this transcription factor led to a reduction of abnormal *α*-synuclein expression (1 μmol SF) and decrease in dopaminergic neuron degeneration in the substantia nigra pars compacta (0.1% GR for 30 days) (78). Further, GR intake (0.1% GR diet for 28 days) protected against the reduction in dopamine transporter density in mice treated with MPTP, indicating its potential in preserving dopaminergic function (86). SF and erucin (from *Eruca sativa* or arugula) at 5 μmol/kg for 4 weeks exhibited neuroprotective effects by protecting against neuronal death and increasing glutathione and Nrf2 levels in a 6-hyzdroxydopamine (6-OHDA) PD mouse model (87). Finally, two synthetic ITC protected dopaminergic neurons and prevented motor deficits associated with PD in mice (88, 89).

Three *in vitro* studies were also identified, providing mechanistic insights into the neuroprotective effects of SF. The first showed that SF treatment (56.4  μmol/kg/day for 10 days) upregulated Nrf2 and BDNF while downregulating MeCP2 in SH-SY5Y cells (84). A second study reported that compared to erucin, SF (5 μmol/kg for 4 weeks) exhibited greater Nrf2 activation, glutathione levels, and resistance to 6-OHDA-induced apoptosis in SH-SY5Y cells (87). The third study revealed that Nrf2 activation by SF (1  μmol SF) suppressed CCAAT/enhancer-binding protein *β* (C/EBPβ) transcription in SH-SY5Y cells treated with MPP+ (1-methyl-4-phenylpyridinium) (85). SF also reduced *α*-synuclein aggregation in HEK293-α-Syn-YFP cells treated with preformed fibrils. These findings collectively draw attention to the role of SF in antioxidant defense, neurotrophic support, and neuroprotection.

Additional studies have highlighted the neuroprotective effects of other natural compounds, such as benzyl ITC and indole-3-carbinol (not an ITC, but derived from indole glucosinolates), in preclinical models of PD. Benzyl ITC (67.1 μmol/kg/day for 12 weeks) enhanced the activity and expression of an endogenous antioxidant, GST-*π* (glutathione S-transferase pi), in Wistar rats with zinc-induced Parkinsonism and ameliorated neurological deficits, neurodegenerative markers, and oxidative stress (90). Similarly, indole-3-carbinol (680 μmol/kg/day) amended striatal dopamine levels and neurodegeneration, and prevented motor dysfunctions in rotenone-induced PD in male albino rats (91).

### 3.3 Multiple sclerosis

Multiple sclerosis (MS) is an inflammation-mediated demyelinating disease of the human central nervous system, with neurodegeneration being the primary cause of irreversible neurological disability. A total of 5 *in vivo* studies have demonstrated the benefits of ITC in MS.

The experimental mouse models of autoimmune encephalomyelitis (EAE) and cuprizone (a copper-chelator causing demyelination) are the most commonly employed animal models used to study different aspects of MS pathology. In one study using the EAE model, invesigators demonstrated that SF (282 μmol/kg/day for 14 days before EAE induction and 28 days after EAE induction) had anti-inflammatory effects, improved clinical scores in behavioral study, and inhibited demyelination in the spinal cords (92). Four additional studies have reported protective effects of *Moringa oleifera* (or its ITC moringin) in animal models of MS. First, mice receiving moringin pretreatment (16.4 μmol/kg glucomoringin +5 μL myrosinase daily for a week before EAE induction and 28 days after EAE induction) exhibited increased expression of Nrf2, reduced cell apoptosis, and suppressed aberrant Wnt-*β*-catenin signaling in EAE mice (93). Another study demonstrated that *Moringa oleifera* (1.875 mg/mL for 5 weeks) reversed cuprizone-induced neuropathological deficits by mitigating oxidative stress, memory decline, and cortico-hippocampal neuronal deficits in Wistar rats (94). Moreover, *Moringa oleifera* leaf extract (1.875 mg/mL/day for 5 weeks) ameliorated cuprizone-induced weight loss and histological alterations in the hippocampal CA3 region in female Wistar rats (95). Co-administration of cuprizone with *Moringa oleifera* (1.875 mg/mL/day for 5 weeks) resulted in improved cellular assortment and delineated cytoarchitectural manifestations in cuprizone-treated rats (95). Finally, a topical treatment of 2% moringin cream twice per day alleviated neuropathic pain in the EAE model by attenuating proinflammatory cytokines (interleukin-17 and interferon-*γ*) while increasing the expression of the anti-inflammatory cytokine interleukin-10 (IL-10) (96).

### 3.4 Amyotrophic lateral sclerosis

Amyotrophic lateral sclerosis (ALS) is a progressive neurodegenerative disease characterized by the degeneration and death of motor neurons, leading to the inability to control muscle movement. One *in vivo* study has examined the effect of glucomoringin on ALS. In a transgenic rat model, daily treatment with glucomoringin (16.4 μmol/kg) + myrosinase (20 μL/rat) for 2 weeks prior to disease onset demonstrated protective effects, as indicated by measuring inflammatory and apoptotic markers, and it inhibited motor neuron degradation, a key to ALS pathophysiology (97).

### 3.5 Huntington’s disease

Huntington’s disease, an autosomal dominant neurodegenerative disorder, involves motor dysfunction, psychiatric issues, and cognitive decline. Mitochondrial dysfunction is a molecular feature implicated in most neurologic disorders, including Huntington’s disease. One *in vivo* study showed that SF (two doses of 28.2 μmol/kg/day) restored mitochondrial function against quinolinic acid-induced damage in an experimental rodent model of Huntington’s disease (98).

### 3.6 Friedreich’s ataxia

Friedreich’s ataxia (FRDA) is a progressive neurodegenerative disease characterized by oxidative stress and mitochondrial dysfunction with decreased expression of the mitochondrial protein frataxin (FXN). Two preclinical studies, both conducted *in vitro*, have examined the effects of SF in FRDA fibroblasts. First, treatment with SF (5 μM for 24 h) increased frataxin protein expression, promoted axonal re-growth in frataxin-silenced motor neurons and restored Nrf2 transcriptional activity in fibroblasts from patients with FRDA (99). In the second study, there was increased expression of FXN and Nrf2 target genes and increased glutathione concentration in SF (10 μM for 24 h) treated FRDA fibroblasts compared to untreated cells (100).

### 3.7 Fragile X-associated tremor/ataxia syndrome

Fragile X-Associated Tremor/Ataxia Syndrome (FXTAS), typically caused by a premutation in the FMR1 (fragile X messenger ribonucleoprotein 1) gene, is a nervous system disorder in older adults characterized by tremors, ataxia, memory issues, and mood disorders. One clinical study (Table 4) and one *in vitro* study have demonstrated the effects of SF on FXTAS. The clinical study was a 24-week open-label trial in 11 adults aged 60–88 years with FXTAS (101). SF (100 μmol SF/day) improved spatial working memory but did not improve other clinical outcomes, molecular changes in mitochondria-derived vesicles, or bioenergetics in PBMCs (101). The *in vitro* study was conducted in FRDA fibroblasts in which SF treatment (5 μmol for 72–96 h) that resulted in improvements in deficient pathways associated with FXTAS in both Nrf-2-dependent and independent manners (102). This was observed in primary fibroblasts from FXTAS-affected subjects ranging from stages 2.5 to 5. These improvements included the modulation of oxidative stress, enhancement of mitochondrial function, promotion of DNA repair, and increased clearance of damaged organelles and macromolecules through parkin-ubiquitin proteasomal mechanisms (102).

**Table 4:** A clinical study reported the effects of glucoraphanin + myrosinase compound on cognitive outcomes in individuals with FXTAS.

Author, Year (Study type)	Study population	Compound(s) delivered	Treatment and dose	Duration of intervention	N	Results
Santos et al., 2023 (94) (Single arm clinical trial)	Adults aged 60–88 with FXTAS	GR + myrosinase	Started with 1 Avmacol tablet and increased every other day by 1 tablet to 6 tablets/day (dosed at 222 μmol GR; equivalent to 100 μmol SF) per day; the highest tolerable dose maintained if intolerant to the maximum amount	24 weeks	11	Improvement in spatial working memory score (*p* = 0.048). No improvement in MoCA scores and molecular changes in mitochondria-derived vesicles.

FXTAS, Fragile-X-Associated Tremor and Ataxia Syndrome; GR, glucoraphanin; SF, sulforaphane; N, number of participants; μmol, micromoles; MoCA, Montreal cognitive assessment.

## 4 Other brain-related disorders

Neurodevelopmental and neurodegenerative conditions share common pathological mechanisms, encompassing neuronal cell death, microglial activation, neuroinflammation, disrupted redox homeostasis, mitochondrial dysfunction, and synaptic dysfunction. There are other brain-related disorders and conditions that also share these mechanisms, including spinal cord injury, traumatic brain injury, diabetes-induced cognitive decline, depression, and anxiety. There is both clinical and preclinical evidence of amelioration of symptoms with ITC treatment for these conditions.

### 4.1 Spinal cord injury

Spinal cord injury (SCI) is marked by neurologic dysfunction and neuronal death resulting from inflammatory and oxidative insults. Seven *in vivo* studies with ITC treatment for SCI have been identified.

SF has shown notable effects in mitigating inflammation and oxidative stress associated with SCI in mouse models. SF treatment (56.4 μmol/kg for 11 days) increased the expression of Nrf2 and phase 2 cytoprotective enzymes while inhibiting markers induced by inflammation in two mouse models when inflammatory pain was induced in the spinal cord using complete Freund’s adjuvant (103). Beyond its anti-inflammatory effects, SF enhanced the antinociceptive actions of morphine, increasing the effectiveness of pain alleviation compared to morphine alone (103). Similarly, rats subjected to mild thoracic SCI had significantly greater levels of Nrf2 and glutamate-cysteine ligase, decreased levels of inflammatory cytokines and spinal cord lesion volumes, and improved coordination after SCI with an SF dose of 28.2 μmol/kg/day for 3 days (104). SF (28.2 μmol/kg an hour after SCI induction) activation of Nrf2 post-SCI improved locomotor function and reduced inflammatory damage, neuron death, and spinal cord edema, while neurological deficits were more severe and the protective effect of SF was not observed in Nrf2 deficient mice (105). SF (282 μmol/kg at 10 min and 72 h after SCI contusion) in a rat model of contusion SCI upregulated antioxidant responses, reduced inflammatory cytokine mRNA levels, improved hindlimb locomotor function, and increased serotonergic axons caudal to the site of lesion (106). Further, histological examination of spinal cord sections revealed improvements in demyelination and apoptosis, but not inflammation as compared to the vehicle group with SFX-01 (10, 50 or 300 mg/kg twice a day for 3 weeks), a stabilized form of SF, treatment in EAE mice (107).

In addition to SF, moringin has shown promising anti-inflammatory and anti-apoptotic effects in the context of SCI. Pretreatment of gingival mesenchymal stem cells (GMSCs) with moringin (0.05 μM 1 h post-SCI) in a mouse model of SCI demonstrated anti-inflammatory and anti-apoptotic effects (108). Moringin-treated GMSCs reduced inflammatory marker levels, restored spinal cord morphology, increased expression of anti-apoptotic marker B-cell lymphoma 2 (Bcl-2) and decreased expressions of apoptotic markers such as Bcl-2-associated X protein (Bax) and caspases 3 and 9 (108). Similarly, post-injury administration of moringin (16.4 μmol glucomoringin/kg + 5 μL myrosinase/day for 7 days before SCI and continued until sacrifice) in mice subjected to SCI through the application of vascular clips showed protective effects against secondary damage by reducing oxidative stress, inflammation, and apoptosis (109).

### 4.2 Traumatic brain injury

Traumatic brain injury (TBI), often resulting from blunt force, shares pathophysiological similarities with stroke-induced cerebral ischemia, involving cellular damage from excitotoxicity, oxidative stress, apoptosis, and inflammation. Preclinical studies including eight *in vivo* and one *in vitro* experiments have been identified to examine the effects of ITC on traumatic brain injury.

SF has consistently demonstrated protective effects against cerebral ischemia/reperfusion (CIR) injury and associated inflammation in rat models. Two rat studies using a CIR model found SF (28.2 μmol or 56.4 μmol /kg 1 h at the beginning of the injury; 56.4, 112.8 or 225.6 μmol /kg/day for 14 days) had congruent protective effects against CIR-induced damage and inflammation (110, 111). Additionally, SF demonstrated protective effects in rodent models of vascular cognitive impairment induced by ischemia at 56.4 μmol/kg twice a week (112), subarachnoid hemorrhage at 282 μmol/kg every 24 h (113), and intracerebral hemorrhage at 5.64 μmol/kg twice a day (114), with upregulation of Nrf2 and reduced inflammation reported in the latter two.

Moreover, studies have investigated the potential synergistic effects of SF with other compounds in mitigating neuroinflammation and oxidative stress associated with traumatic brain injury. An *in vivo* study in a rat model of traumatic brain injury demonstrated that a combination therapy of N-acetylcysteine and SF yielded modest improvements in motor movement and coordination (115). However, there were no significant effects on composite neuromotor score or cortical lesion area (115). Yet, an *in vitro* combination therapy with N-acetylcysteine and SF (5 and 10 μM) significantly reduced neuroinflammation and nitrite levels while improving neuronal viability (115).

Similarly, moringin treatment (~8 μmol glucomoringin/ml plus 30 μL enzyme 15 min after beginning of ischemia and daily) of rats with CIR injury prevented CIR-induced damage and decreased inflammatory and oxidative mediators that exacerbate disease progression (116). Further, allyl isothiocyanate (AITC; 101 μmol/kg immediately after TBI induction) administered immediately after traumatic brain injury in mice significantly reduced infarct volume and serum IgG extravasation, revealing an improvement in blood–brain barrier permeability (117). AITC also decreased proinflammatory cytokines while increasing Nrf2, neural outgrowth and regeneration marker growth-associated protein 43 (GAP43), and neurogenesis-associated neural cell adhesion molecule levels (117).

### 4.3 Diabetes-related cognitive decline

Compelling evidence establishes a correlation and potential pathophysiological connection between type 2 diabetes (T2D) and cognitive dysfunction, leading to an increased risk of AD (118). The implicated mechanisms include defects in insulin signaling, neuroinflammation, and mitochondrial metabolism, among others (118). Both SF and moringin have been studied for their potential to attenuate diabetic complications including cognitive decline (119, 120). Eight *in vivo* and two *in vitro* experiments have been identified that demonstrate the effect of these ITC on diabetes-related cognitive decline.

Several studies have investigated the potential neuroprotective effects of SF in various models of diabetes-induced cognitive impairment. In a streptozotocin-induced diabetic rat model, SF (141 μmol/kg/day for 14 days) supplementation yielded preventive effects against learning and memory impairment (121). The rats also showcased attenuated decline in memory, protection against hippocampal neuron apoptosis, decreased caspase-3 expression, and increased expression of an antiapoptotic marker, MCL-1 (myeloid leukemia cell differentiation protein). Similarly, in a type 2 diabetes mellitus mouse model, SF (5.64 μmol/kg/day for 28 days) administration mitigated cognitive decline and reduced pathological hallmarks of AD, such as Aβ-oligomers, Aβ 1–42 plaques, and phospho-tau (122). SF activated Nrf2-regulated antioxidant defenses, enhancing the expression of NrF2 downstream genes HO-1 and NQO1. The study also reported reduced reactive oxygen/nitrogen species in the mice brains and mitigated cognitive decline (122). Additionally, SF (5.64 μmol/kg/day for 15 days) demonstrated therapeutic effects in a rat model induced with nicotinamide and streptozotocin by restoring insulin levels, normalizing motor coordination, improving sensory responses, and ameliorating histopathological changes (123). One study also examined a combination therapy with ulinastatin (10,000 U/kg/day for 26 days) and SF (141 μmol/kg/day for 26 days), which attenuated streptozotocin-induced diabetes and vascular dementia in rats, improving behavioral, endothelial, and biochemical parameters (124).

One *in vitro* study showed that a combination of SF (20 μmoL/L) and vitamin E (200 μg/mL alpha-tocopherol) protected against glucotoxic damages on neuronal structure and function in the nematode *Caenorhabditis elegans*, a model organism used to study the effects of high glucose on neuronal function (125). The duo also prevented reactive oxygen species and methylglyoxal-derived advanced glycation end products formation under hyperglycemic conditions (125). In both *in vitro* (0.5 μM for 24 h) and *in vivo* (141 μmol/kg/day for 14 days) settings, SF mitigated high glucose-induced apoptosis by inhibiting the upregulation of apoptotic markers and the downregulation of antiapoptotic maker Bcl-2 protein in hippocampal neurons exposed to high glucose (126).

*Moringa oleifera* has also been shown to provide neuroprotection against diabetes-induced cognitive dysfunction. In one study, *Moringa oleifera* leaves added to the diet (0.5 g, 1.0 g and 2.0 g per day for 30 days) reduced brain cholinergic enzymes (AChE, BChE), glycemic index, and lipid profile (TC, TG, LDL-C), while elevating antioxidant enzymes (SOD, CAT) in rats compared to controls not fed the leaves (127). In another study, the plant’s leaves (2.0, 4.0 and 8.0 g/kg/day for 8 weeks) exhibited protective effects on cognitive dysfunction and hippocampal neuron apoptosis in streptozotocin-induced diabetic rats (128). Furthermore, hyperglycemic rats supplemented with *Moringa oleifera* leaves or seeds (2 and 4% of the diet for 14 days) exhibited elevated levels of various antioxidant enzymes and glutathione, concomitantly decreasing biomarkers associated with hyperglycemia-induced cognitive decline (129).

### 4.4 Anxiety and depression

Mice subjected to acute or chronic stress often exhibit depressive and anxiety-like behaviors, paralleled by immune dysregulation. One RCT and 13 *in vivo* with ITC treatment have been identified for anxiety and depression.

#### 4.4.1 Clinical study

There has been one randomized, double-blind, placebo-controlled clinical trial on the antidepressant effects of SF (*N* = 66) (130). In patients with a history of cardiac interventions, 169.2 μmol/day SF for 6 weeks alleviated mild to moderate depression, leading to greater improvements in the Hamilton Rating Scale for Depression (HAM-D) compared to placebo (*p* < 0.001). Participants on SF demonstrated greater response to treatment rates at the end of the trial compared to those on placebo (30% vs. 6.67%, *p* < 0.05) (Table 5).

**Table 5:** A clinical study reported the effects of sulforaphane on depression outcomes in cardiac patients with mild to moderate depression.

Author, Year (Study Type)	Study population	Compound(s) delivered	Treatment and dose	Duration of intervention	N	Results
Ghazizadeh-Hashemi, et al., 2021 (RCT)	Cardiac patients (40–65 years old) with mild to moderate depression	SF	169.2 μmol/day	6 weeks	66	Improvement in the HAM-D in the SF group (*p* < 0.001) Greater response to treatment rates in SF than placebo (30% vs. 6.67%, *p* = 0.042) Remission rate not significantly higher in the SF group than placebo (23.33% vs. 3.33%, *p* = 0.052).

RCT, randomized controlled trial; N, number of participants; mg, milligrams; SF, sulforaphane; mg, milligrams; HAM-D, Hamilton Rating Scale for Depression.

#### 4.4.2 Preclinical studies

Preclinical studies have further elucidated the potential mechanisms underlying the antidepressant and anxiolytic effects of SF. SF treatment (56.4 μmol/kg/day for 14 days) significantly reversed anxiety-like behaviors in acutely and chronically stressed mice, reducing serum corticosterone, adrenocorticotropic hormone, interleukin-6, and TNF-*α* in chronically stressed mice (131). SF (16.92, 56.4 and 169.2 μmol/kg) also combated inflammation-induced depression-like behavior in mice, reducing immobility time and restoring dendritic changes induced by LPS administration (132). Similarly, chronic administration of 56.4 μmol/kg SF for 15 days alleviated anxiety-like behavior and produced antidepressant effects in neuropathic mice, as evidenced by behavioral tests in neuropathic mice (133). Moreover, SF (28.2 μmol/kg/day for 7 days) reversed Aβ-oligomer-induced depressive-like behavior in rats, protecting the serotonergic system and mitigating depressive-like behavior (134).

In one study, GR administration (114.17 μmol/kg) brought about antidepressant effects showcased through reduction in immobility frequency in the forced swimming test (FST) and restoring serotonin (5-HT) levels in soluble Aβ 1–42 (a 42-residue form of Aβ) treated rats (135). Further, treating mice with dietary GR (0.1% GR food pellets) during juvenile and adolescent stages has been shown to prevent LPS-induced depression-like behaviors in adulthood (132, 136).

*Moringa oleifera* has also demonstrated promising anxiolytic and antidepressant effects in animal models. For example, ITC-rich extracts of *Moringa oleifera* leaf (500 mg/kg administered 30 min prior to tests) induced anxiolytic effects in Swiss mice in light–dark and open field tests (OFT) (137). Additionally, treatment with *Moringa oleifera* seed oil (1 or 2 mL/kg for 23 days) decreased anxiety-and depression-like behaviors, measured using the OFT and FST in the water-immersion restraint stress mouse model. (138). And, pretreatment with *Moringa oleifera* leaf extract (400 mg/kg/day for 14 days) produced anxiolytic effects in both FST and tail suspension test (TST) in the hepatic encephalopathy mouse model (139).

Other ITC-rich plants have also been studied for their antidepressant and anxiolytic properties. Ethanolic and methanolic extracts of *Camelina sativa* var. Madalina (5 g/kg), rich in sinapine, glucosinolates, and flavonol glycosides, brought about anxiolytic and antidepressant effects in stress-induced irritable bowel syndrome mouse models (140). *Isatis tinctoria* leaf extract (50, 100 or 500 mg/kg) reversed stress-induced anxiety-like behavior and regulation of neurooxidative, neuronitrosative, and neuroimmune pathways in mice (141). Further, *Raphanus caudatus* (250 and 500 mg/kg), a radish variety, significantly reduced anxiety-like behavior in mice, comparable to diazepam, a pharmaceutical anxiolytic drug (142). And, indole-3-carbinol (408 μmol/kg for 10 days), an ITC usually found in cabbage, broccoli, and collard greens, prevented chronic social defeat stress (CSDS)-induced behavioral abnormalities associated with depression in mice, though it did not affect behavioral abnormalities related to anxiety (143).

## 5 Adverse effects

Progoitrin and indole glucosinolates in *Brassica* vegetables can degrade to goitrin in the presence of myrosinase, potentially decreasing thyroid hormone production (144–146). While a clinical study found that a single 25 mg or 50 mg dose of goitrin inhibited thyroid radioiodine uptake, two 10 mg doses did not have this effect (146, 147). Vegetables with low progoitrin levels, such as commercial broccoli and kale, pose minimal risk to iodine uptake. A recent systematic review of 123 studies suggests that regular consumption of Brassica vegetables does not pose a risk of adverse effects, particularly with adequate iodine intake (148).

Some ITCs demonstrate a hormetic effect, offering cytoprotective benefits at low doses and cytotoxic, antitumor properties at higher doses (149, 150). A few studies have shown that SF increases reactive oxygen species (ROS) generation and DNA double-strand breaks (DSB) in cancer cells in a dose-and time-dependent manner (151, 152). While these effects reduce cancer cell survival, normal cells like human dermal fibroblasts and gastric mucosal cells exhibit higher viability and faster DNA repair when exposed to similar doses, suggesting that SF could be an effective cancer therapeutic without harming normal cells (153, 154).

Ingestion of isothiocyanate-containing preparations may cause adverse effects. Higher doses of SF can cause a harsh, burning sensation in the back of the throat, which may deter some from consuming broccoli or sulforaphane preparations (155, 156). Strategies to mask flavors of brocolli extracts and moringa teas have been studied (157, 158). Some individuals may experience gastrointestinal discomfort when consuming SF in large quantities (155). Gradually increasing the dose may help mitigate these effects, allowing the gut to adapt over time (159).

## 6 Alternative treatments

Bioactive compounds, along with plant sources beyond cruciferous vegetables and dietary nutrients, offer potential alternative approaches for managing neurologic disorders (160–162). Among these, polyphenols (163), flavonoids (164) and certain herbs (165) along with other phytonutrients (166) have demonstrated therapeutic benefits in neurologic disorders. Curcumin is one of the most extensively studied compounds (167, 168), with other common treatments including quercetin (169), resveratrol (170) and luteolin (171). Despite the potential of dietary nutrients like vitamin D and omega-3 fatty acids for neuroprotection (172), findings from studies have yielded mixed results without consistent beneficial effects (173, 174). Exploring the effect of nutraceuticals as treatments for neurologic disorders remains a significant area of interest.

## 7 Methods

All research articles were retrieved from PubMed and Scopus. The search criteria consisted of the following terms: (“glucosinolate” OR “glucoraphanin” OR “sulforaphane” OR “isothiocyanate” OR “moringin”) AND (“brain” OR “mental” OR “cognit*” OR “neuro*” OR “psych*”) NOT (FITC OR SITS OR “fluorescein isothiocyanate” OR RITC OR “rhodamine B isothiocyanate” OR “fentanyl isothiocyanate” OR “di-o-tolyl-guanidine-isothiocyanate” OR “isothiocyanate labelled” OR IFN OR “rhodamine isothiocyanate” OR LHRH OR “affinity labeled”) for articles published from November 2018 through May 2024. Since this review extends the previous review published in 2018, literature from that review is included along with additional relevant clinical trials that were omitted in the original publication. After 404 duplicates were removed, 1,108 abstracts were screened by two independent reviewers, and 293 full texts were reviewed. A total of 106 articles (clinical and preclinical studies) were included. Criteria for inclusion were original research articles focusing on isothiocyanates derived from natural plant sources and their effects on neuropsychiatric disorders and other brain-related conditions. Studies focusing on other phytochemicals like flavonoids, alkaloids, and polyphenols, and non-brain-related outcomes, were excluded.

## 8 Conclusion

We illustrate that cruciferous and related vegetables (or supplements rich in glucosinolates or ITC) can contribute to a dietary strategy for combating neuropsychiatric disorders and improving the quality of life. A few studies have also reported their effects as comparable to pharmaceutical treatments with fewer side effects. While these plants contain various phytochemicals, it is the potent and highly bioavailable compounds within the glucosinolate/myrosinase/ITC system that uniquely contribute to the highly potent indirect antioxidant properties of these vegetables. However, other phytochemicals in these plants, including phenols and flavonoids, have also been shown to be beneficial for brain-related outcomes (175). It may therefore be more valuable to consume the vegetable as a whole (or as sprouts) or as water extracted vegetable supplements rather than those containing only a high refined glucosinolate or isothiocyanate.

Through our comprehensive search, we found around 11 preclinical studies on the effects of ITCs from other cruciferous vegetables including maca, radish, and wasabi on neuropsychiatric disorders. A realm of opportunity remains to explore the potential benefits of cabbage, turnips, mustard and other cruciferous vegetables on neuroinflammation and outcomes related to neuropsychiatric disorders.

While this review updates our review published 6 years ago (26), it does not delve into the extensive epidemiological evidence supporting all the benefits of ITC. Furthermore, it is essential to note that there is a plethora of literature highlighting their other properties (155). The comprehensive understanding of the underlying mechanisms now sheds light on why this protection extends across a broad spectrum of chronic conditions, encompassing both neurodevelopmental and neurodegenerative origins. This emphasizes the potential of cruciferous and other ITC-containing vegetables as a safe and effective dietary intervention for enhancing brain health and mitigating the risk and effects of various brain-related disorders. Given the increasing number of *in vitro* and animal studies that show the metabolic and behavioral benefits and safety of ITC, it is now important that clinical trials be expanded to facilitate their application in patients with disorders of the nervous system.
